# Advanced computational tools, artificial intelligence and machine-learning approaches in gut microbiota and biomarker identification

**DOI:** 10.3389/fmedt.2024.1434799

**Published:** 2025-04-15

**Authors:** Tikam Chand Dakal, Caiming Xu, Abhishek Kumar

**Affiliations:** ^1^ Genome and Computational Biology Lab, Department of Biotechnology, Mohanlal Sukhadia University, Udaipur, India; ^2^ Beckman Research Institute of City of Hope, Monrovia, CA, United States; ^3^ Department of General Surgery, The First Affiliated Hospital of Dalian Medical University, Dalian, China; ^4^ Manipal Academy of Higher Education (MAHE), Manipal, India; ^5^ Institute of Bioinformatics, International Technology Park, Bangalore, India

**Keywords:** gut microbiome, gut microbiota, artificial intelligence (AI), machine learning (ML), network-based methods, biomarker discovery, precision medicine, personalized treatment

## Abstract

The microbiome of the gut is a complex ecosystem that contains a wide variety of microbial species and functional capabilities. The microbiome has a significant impact on health and disease by affecting endocrinology, physiology, and neurology. It can change the progression of certain diseases and enhance treatment responses and tolerance. The gut microbiota plays a pivotal role in human health, influencing a wide range of physiological processes. Recent advances in computational tools and artificial intelligence (AI) have revolutionized the study of gut microbiota, enabling the identification of biomarkers that are critical for diagnosing and treating various diseases. This review hunts through the cutting-edge computational methodologies that integrate multi-omics data—such as metagenomics, metaproteomics, and metabolomics—providing a comprehensive understanding of the gut microbiome's composition and function. Additionally, machine learning (ML) approaches, including deep learning and network-based methods, are explored for their ability to uncover complex patterns within microbiome data, offering unprecedented insights into microbial interactions and their link to host health. By highlighting the synergy between traditional bioinformatics tools and advanced AI techniques, this review underscores the potential of these approaches in enhancing biomarker discovery and developing personalized therapeutic strategies. The convergence of computational advancements and microbiome research marks a significant step forward in precision medicine, paving the way for novel diagnostics and treatments tailored to individual microbiome profiles. Investigators have the ability to discover connections between the composition of microorganisms, the expression of genes, and the profiles of metabolites. Individual reactions to medicines that target gut microbes can be predicted by models driven by artificial intelligence. It is possible to obtain personalized and precision medicine by first gaining an understanding of the impact that the gut microbiota has on the development of disease. The application of machine learning allows for the customization of treatments to the specific microbial environment of an individual.

## 1 Introduction

The gastrointestinal tract, also referred to as the gut, has a large and intricate ecosystem filled with billions of bacteria. The gut microbiome is a complex community consisting of a wide variety of bacteria, archaea, fungus, and viruses (1). Each of these components has an important role in preserving human health. The human microbiome, composed of diverse bacteria, has a vital role in the metabolic processes required for the proper functioning of enzymes in the gut mucosa and liver, as well as the overall metabolism of the host (2). The makeup of this collection of microorganisms is not fixed; it consistently changes over the course of our lives, influenced by several factors such as diet, lifestyle, environment, and even heredity. The gut microbiota influences the host's well-being via altering the biochemical makeup of the diet. A study has been carried out to investigate the functions of various bacteria in metabolic pathways, namely in the breakdown of food components, because of the crucial role of gut microbiota in human immune system (3, 4).

The human microbiome, a diverse collection of microorganisms residing in various anatomical sites, plays a crucial role in health and disease (Table 1). Microorganisms within the human body may engage in commensal, mutualistic, or harmful relationships, influencing host physiology through the production of various metabolites (10). Traditional culture-based methods have historically limited our understanding of these complex microbial communities. However, advancements in metagenomics (MGs) have significantly expanded our ability to identify and characterize previously unknown microbial species and their functions, particularly through whole genome sequencing (WGS) and marker gene sequencing (11). These technologies have been instrumental in large-scale projects like the Human Microbiome Project (HMP) and the American Gut Project, generating extensive datasets that have deepened our understanding of host-microbiome interactions (12).

**Table 1:** Gut microbes and metabolites: systemic manifestations linked to several Gut associated diseases and disorders.

Condition	Key findings	Microbial changes	Mechanisms	References
Obesity	Obesity linked to an increase in specific gut microbiota; global prevalence of obesity has increased significantly over the last 40 years.	Increased *Firmicutes, Bacteroidetes, Rhizobium, Lactococcus, Clostridium*	Production of short-chain fatty acids (SCFAs) like butyrate increases energy supply to the host, promoting weight gain.	(5)
Type 2 diabetes	Dysbiosis associated with poor glucose tolerance, insulin resistance, and systemic inflammation.	Altered gut microbiota composition affecting butyrate production	Gut microbiota influences glucose metabolism, insulin signaling, and inflammation.	(6)
Cardiovascular disease	Gut dysbiosis linked to coronary artery disease and hypertension.	Increased *Collinsella, Lactobacilli, Escherichia-Shigella*; decreased *Roseburia, Eubacterium spp.*	Dysbiosis affects cholesterol metabolism, promotes TMAO production which contributes to atherosclerosis, and alters bile acid metabolism.	(7)
Cancer	Gut dysbiosis linked to colorectal cancer, hepatocellular carcinoma, gastric cancer, breast cancer, and prostate cancer.	Presence of pro-inflammatory and genotoxic bacteria	Bacteria produce cytotoxic and genotoxic metabolites that damage DNA, promote tumorigenesis, and influence tumor progression.	(8)
Neurological disorders	Dysbiosis potentially linked to depression, anxiety, Alzheimer's disease, Parkinson's disease, multiple sclerosis, and autism spectrum disorders.	Changes in gut microbiota composition influencing gut-brain axis	Dysbiosis disrupts communication between the gut and brain, potentially affecting CNS development and function.	(9)

The study of the gut microbiome has seen significant advancements in recent years, with the development of a variety of computational tools and techniques that have revolutionized the field (13). The advent of next-generation sequencing technologies has enabled the comprehensive profiling of microbial communities, allowing researchers to uncover the vast diversity and complexity of the gut microbiome (14). Metagenomics approaches, which involve the sequencing of genetic material extracted directly from environmental samples, have become a cornerstone of gut microbiome research, providing a wealth of information on the taxonomic composition and functional capabilities of these microbial communities (15, 16).

In the past, gut microbiome research has relied heavily on traditional methods such as culture-based techniques and phylogenetic marker gene analysis, notably 16S rRNA sequencing. These approaches have provided foundational knowledge, allowing researchers to identify and classify microbial taxa within complex communities (17). However, traditional methods have significant limitations, particularly in terms of resolution and depth. Culture-based techniques are limited by their inability to grow the vast majority of gut microorganisms, while 16S rRNA sequencing offers limited taxonomic resolution and does not provide functional insights into microbial activities (18).

Advanced computational and multi-omics approaches are transforming gut microbiome research by enabling a deeper exploration of microbial functions beyond traditional taxonomic classifications. These approaches link microbial composition with potential roles in health and disease, offering a more comprehensive understanding of the microbiome's functional capacities (19). Visualization and statistical techniques play a crucial role in interpreting vast datasets, allowing researchers to identify patterns and correlations within microbiome data. By focusing on the small molecules and proteins produced by the microbiome, metabolomics and metaproteomics provide direct insights into microbial activity and its impact on host physiology (20).

Technological advancements have significantly enhanced the study of metabolomes and transcriptomes, deepening our understanding of microbial gene expression and function. Network analysis and machine learning further enrich this field by uncovering complex microbial interactions and predicting potential biomarkers and therapeutic targets (21). As the integration of multi-omics data with AI and machine learning continues to evolve, these approaches are poised to unlock new insights into the gut microbiome, paving the way for advancements in personalized medicine and novel therapeutic strategies (22).

In addition to computational tools, the field of gut microbiome research has also benefited from the integration of multi-omics techniques, such as metatranscriptomics, meta-proteomics, and metabolomics (23). These approaches provide a more comprehensive understanding of the gut microbiome by capturing not only the taxonomic composition, but also the functional activities, metabolic processes, and interactions within the microbial community (24).

Furthermore, the integration of artificial intelligence and machine learning algorithms has opened up new frontiers in gut microbiome research. These advanced analytical techniques have the potential to uncover complex patterns and associations within the gut microbiome, enabling the identification of novel biomarkers and the development of predictive models for various health and disease states (25, 26).

## 2 Traditional methods for gut microbiome research and their limitation

The study of the gut microbiome has become an increasingly important field in recent years, as researchers have come to recognize the critical role that the diverse community of microorganisms inhabiting the human gastrointestinal tract plays in maintaining overall health and contributing to various disease states (27–29). The advancement of molecular techniques, particularly next-generation sequencing technologies, has revolutionized our ability to characterize the composition, function, and ecology of the gut microbiome in unprecedented detail (30).

### 2.1 Key molecular techniques for microbiome analysis and their applications

#### 2.1.1 Quantitative real-time polymerase chain reaction (qPCR)

qPCR is a powerful tool in microbiome analysis that allows for the quantification of specific DNA sequences. It is used to measure the abundance of particular microbial taxa or genes within a sample, providing precise and sensitive data on microbial population dynamics. This technique is especially valuable in monitoring the effects of environmental changes, treatment interventions, or disease conditions on microbial communities (31).

#### 2.1.2 Denaturing gradient gel electrophoresis (DGGE)

DGGE is used to separate DNA fragments based on their sequence-specific melting behaviour. In microbiome analysis, DGGE allows researchers to profile microbial community diversity by comparing the band patterns generated from different samples. This technique is particularly useful for detecting shifts in microbial populations and identifying dominant species or variants in complex communities (32).

#### 2.1.3 Terminal restriction fragment length polymorphism (T-RFLP)

T-RFLP is a molecular fingerprinting technique used to analyse the diversity of microbial communities. It involves the digestion of amplified DNA with restriction enzymes, followed by the separation of terminal fragments by size. The resulting fragment patterns reflect the community composition, allowing researchers to compare microbial diversity across samples and assess the impact of various factors on community structure (33).

#### 2.1.4 Fluorescence *in situ* hybridization (FISH)

FISH is a technique that uses fluorescent probes to target specific DNA or RNA sequences within microbial cells. In microbiome analysis, FISH enables the visualization and identification of specific microorganisms within their natural environment, often in conjunction with microscopy. This technique is particularly useful for studying the spatial distribution of microbes, understanding microbial interactions, and linking microbial identity to function within a community (34).

### 2.2 Limitations of molecular microbiome analysis techniques

Quantitative Real-Time Polymerase Chain Reaction (qPCR) is highly specific but may not capture the full microbial diversity due to its reliance on primers targeting specific sequences, potentially missing out on less abundant or uncharacterized taxa. Denaturing Gradient Gel Electrophoresis (DGGE) can resolve differences in microbial communities but often lacks sensitivity for detecting subtle variations and may not accurately reflect community composition due to issues with fragment resolution and band intensity interpretation. Terminal Restriction Fragment Length Polymorphism (T-RFLP) provides a fingerprint of microbial diversity but can suffer from inconsistencies in fragment size due to variability in restriction enzyme activity and PCR amplification, which may affect reproducibility. Fluorescence *in situ* Hybridization (FISH) offers detailed spatial information but is limited by the availability of specific probes and the potential for non-specific binding, which can complicate the interpretation of microbial distribution and interactions. Each method's limitations necessitate complementary approaches and careful interpretation to obtain a comprehensive understanding of microbiome dynamics.

### 2.3 Traditional methods: phylogenetic marker gene analysis and sequencing

One of the primary tools utilized in gut microbiome research is marker-gene analyses, which profile the microbial community by sequencing specific genetic markers, such as the 16S ribosomal RNA gene (35). This approach provides information about the taxonomic composition of the microbiome, allowing researchers to identify the dominant bacterial phyla and track changes in community structure across different populations or conditions. While these surveys offer valuable insights, researchers are now transitioning to integrate other data types, such as metabolite, metaproteome, or metatranscriptome profiles, to gain a more comprehensive understanding of the gut microbiome and its functionality (36).

Marker-gene surveys: These approaches profile the microbial community by targeting and sequencing specific marker genes, such as the 16S rRNA gene, which provide varying degrees of taxonomic specificity and phylogenetic information. Disease states (26, 27, 37). The incorporation of these multi-omics approaches has been instrumental in advancing our understanding of the gut microbiome and its role in human health and disease (26).

One of the most commonly used marker genes is the 16S rRNA gene, which provides valuable taxonomic specificity and phylogenetic information. The 16S rRNA gene is highly conserved among bacteria but contains variable regions that allow for the identification and classification of bacterial taxa at various levels of resolution. This gene is particularly useful for assessing microbial diversity and community composition in various environments.

Shotgun metagenomics: This approach involves the sequencing of the entire genomic content of the microbial community, providing a deeper understanding of the functional potential of the gut microbiome, including the identification of specific genes and pathways involved in various metabolic processes (38).

Through the use of these diverse tools and techniques, researchers have gained valuable insights into the gut microbiome and its complex interactions with the host, paving the way for the development of novel diagnostic and therapeutic interventions for a wide range of health conditions (26, 37, 39).

### 2.4 Limitations of traditional microbiome analysis techniques

While marker-gene surveys have been widely used in gut microbiome research, they have several limitations. The choice of the specific marker gene, the DNA extraction protocol, and the sequencing platform can all introduce biases that can lead to inconsistencies in the observed microbial community composition. Additionally, marker-gene surveys often lack the resolution to fully capture the functional diversity of the gut microbiome, as they primarily provide information about the taxonomic structure rather than the metabolic and functional capabilities of the microbial community (40).

Previously, gut microbiome analysis relied on 16S ribosomal RNA (rRNA) gene sequencing. This method targets a specific section of the 16S rRNA gene, a genetic indication found in all bacteria. It determines the identity and percentage of bacterial species in a sample. 16S rRNA sequencing has helped us understand the gut microbiome, but it has limits. First and foremost, this method provides limited gut bacteria functional data. It offers information about gastrointestinal residents but not their activities or behaviours. Due to its focus on bacteria, 16S rRNA sequencing may not capture the whole range of intestinal microbes (41).

To address these limitations, researchers have turned to more comprehensive approaches, such as shotgun metagenomics, which involve the sequencing of the entire genomic content of the microbial community. Shotgun metagenomics can provide a deeper understanding of the functional potential of the gut microbiome, as it allows for the identification of specific genes and pathways involved in various metabolic processes. However, the analysis of shotgun metagenomics data can be computationally intensive and requires specialized bioinformatics expertise (35).

In addition to computational tools, the field of gut microbiome research has also benefited from the integration of multi-omics techniques, such as metatranscriptomics, meta-proteomics, and metabolomics. These approaches provide a more comprehensive understanding of the gut microbiome by capturing not only the taxonomic composition, but also the functional activities, metabolic processes, and interactions within the microbial community. The integration of these multi-omics techniques has enabled researchers to unravel the complex relationships between the gut microbiome and various health and disease states, leading to the identification of novel biomarkers and the development of predictive models for person. Bioinformatics platforms: Software such as QIIME, Mothur, and DADA2 are used for processing and analysing sequencing data.

## 3 Computational tools and multi-omics techniques in microbiome analysis

**Figure 1:**
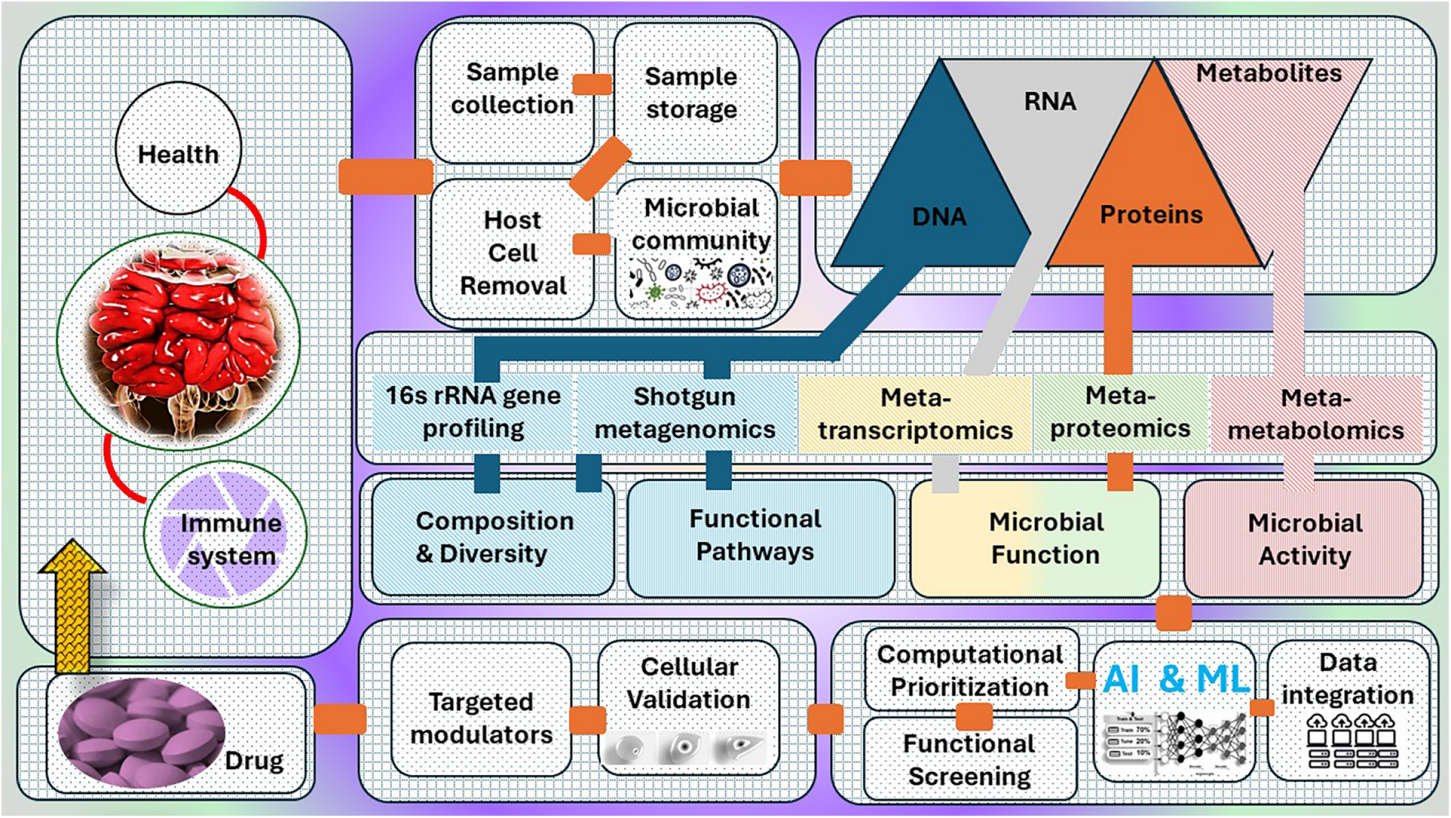
A systematic approach for microbiome data analysis, covering the steps from raw reads to community analyses, incorporating multi-omics techniques and statistical models that allow for more accurate phenotypic and functional profiling.

The conventional techniques employed to examine the gut microbiome, however helpful, provide only a limited understanding of this intricate ecology. In order to gain a comprehensive understanding of the complex relationship between gut bacteria and their influence on human health, a more comprehensive approach is necessary. This is where the potential of multi-omics is harnessed. Multi-omics involves combining data from many biological fields, including metagenomics, metatranscriptomics, and metabolomics, to provide a thorough comprehension of a biological system (Figure 1). Within the framework of the gut microbiome, this method entails examining different forms of “omic” data, each offering a unique viewpoint on the gut environment.

Computational platforms, such as QIIME, Mothur, and DADA2, are widely used to process and analyse sequencing data in gut microbiome studies (26). These marker-gene surveys target and sequence specific genetic markers, like the 16S rRNA gene, which provide varying degrees of taxonomic resolution and phylogenetic information about the microbial community (27, 37). In contrast, shotgun metagenomics involves sequencing the entire genomic content of the microbial community, enabling a more comprehensive understanding of the functional potential of the gut microbiome, including the identification of specific genes and metabolic pathways (42). The integration of multi-omics techniques, including metatranscriptomics, metaproteomics, and metabolomics, has been instrumental in advancing our knowledge of the gut microbiome and its complex interactions with human health and disease (43).

A key tool in this field is the QIIME 2 platform, an open-source, community-developed software suite that enables reproducible, interactive, scalable, and extensible microbiome data science (26). QIIME 2 provides a flexible and powerful framework for analysing and visualizing microbiome data, allowing researchers to perform a wide range of analyses, from taxonomic classification to functional profiling, while ensuring the reproducibility and transparency of their research (25, 26). QIIME 2 offers a range of features, including the ability to process and analyse high-throughput sequencing data, perform taxonomic classification, and generate visualizations to aid in the interpretation of results. Additionally, the platform's modular design allows for the integration of various plug-ins, enabling researchers to extend its capabilities to address specific research questions.

The utility of QIIME 2 has been demonstrated in numerous studies, such as the work by Bolyen et al. (26), which described the platform's ability to facilitate reproducible, interactive, and scalable microbiome data analysis (26). Furthermore, the QIIME 2 tutorial by Gonzalez et al. illustrates how the platform can be used for end-to-end analysis of diverse microbiome datasets, including the integration of public data through the Qiita platform (26, 44).

Alongside QIIME 2, other computational tools have also emerged as invaluable resources in gut microbiome research. Algorithms for sequence clustering, taxonomic assignment, and functional prediction have become increasingly sophisticated, allowing researchers to gain deeper insights into the structure and function of gut microbial communities (25, 26).

## 4 Multi-omics techniques in gut microbiome analyses

**Figure 2:**
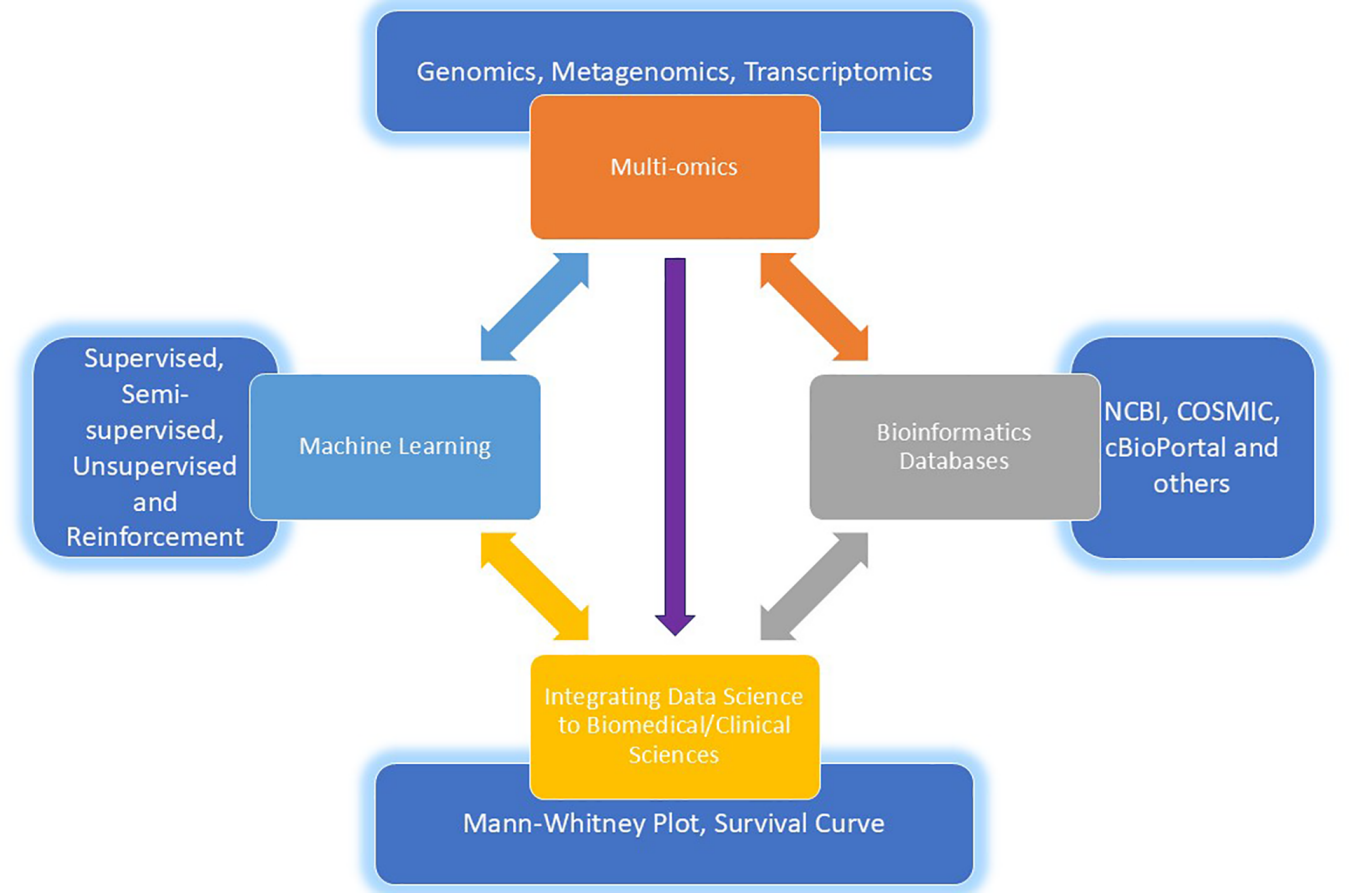
A comprehensive and fascinating intersection of multi-omics, machine learning, databases and the gut microbiota showing evident synergy between multi-omics and machine learning that hold immense promise for advancing our understanding of the gut microbiota and its impact on human health.

In addition to computational tools, gut microbiome research has also greatly benefited from the integration of multi-omics approaches, which provide a more comprehensive understanding of the microbial community and its interactions with the host (Figure 2) (20).

One such approach is metatranscriptomics, which involves the sequencing of the RNA molecules expressed by the microbial community. By analysing the metatranscriptome, researchers can gain insights into the functional activities and gene expression patterns of the gut microbiome, revealing how the microbial community responds to changes in the environment or the host's physiology (45).

Another powerful technique is metaproteomics, which focuses on the identification and quantification of the proteins expressed by the gut microbiome (46). This approach can provide valuable information about the metabolic activities and functional capabilities of the microbial community, as well as the interactions between the microbiome and the host (47).

Furthermore, metabolomics, the study of small-molecule metabolites, has emerged as a crucial tool in gut microbiome research. By analysing the metabolic profiles of the gut microbiome, researchers can uncover the complex interplay between the microbial community and the host's physiology, identifying metabolic pathways and biomarkers that are associated with various health and based on the sources provided, this research paper discusses the different tools and techniques used in gut microbiome studies, including marker-gene surveys, shotgun metagenomics, and multi-omics approaches such as metatranscriptomics, metaproteomics, and metabolomics (48).

The paper highlights the limitations of traditional marker-gene surveys, which can introduce biases and lack the resolution to fully capture the functional diversity of the gut microbiome. To address these limitations, researchers have turned to more comprehensive approaches, such as shotgun metagenomics, which can provide a deeper understanding of the functional potential of the gut microbiome (49).

The integration of multi-omics techniques, including metatranscriptomics, metaproteomics, and metabolomics, has enabled researchers to unravel the complex relationships between the gut microbiome and various health and disease states, leading to the identification of novel biomarkers and the development of predictive models for personalized medicine (50).

### 4.1 Microbiomics

As discussed earlier, this field focuses on the identification and characterization of the microbial population within a sample. Techniques like 16S rRNA sequencing and shotgun metagenomics are employed to assess the composition and diversity of the gut microbiome. Shotgun metagenomics, unlike 16S sequencing, provides a more detailed picture by directly sequencing all the microbial DNA present in a sample, allowing for the identification of not just bacteria but also archaea, fungi, and viruses. Metagenomics refers to the direct examination and analysis of the genetic material present in genomes obtained from diverse sources (51). The term “metabolomics” is often applied incorrectly to 16S rRNA gene sequencing. Sequencing of 16S rRNA is gene-specific and does not examine the entire genome. On the other hand, metagenomics is an approach that utilizes a comprehensive shotgun sequencing methodology to analyze the genetic material of microbes discovered in the environment—without requiring culturing (16, 52). Metagenomics offers an exhaustive enumeration of all microorganisms present in complex environmental samples, including those that are both familiar and unfamiliar and those that are not amenable to laboratory cultivation. In contrast to unimodal phylogenetic studies, which concentrate on the diversity of a solitary gene (e.g., the 16S rRNA gene), metagenomics investigates the multifarious genetic constituents present within microbial communities. Consequently, metagenomics provides a more comprehensive compilation of genomic information and a more precise taxonomic classification (53, 54). The correlation between function and phylogeny is facilitated by genomics, along with the compilation of evolutionary profiles that depict the structure of the microbial community. Significantly, it additionally facilitates the identification of viruses that are challenging to detect through a single-gene approach due to their broad genetic variability and the difficulty in differentiating shared genetic attributes (55). Modern Next-Generation Sequencing (NGS) has progressively replaced traditional Sanger sequencing as the predominant technique for shotgun sequencing in metagenomics over the past few years. In numerous contexts, the 454/Roche and Illumina/Solexa technologies were utilized extensively to analyze metagenomics materials (56). Scientists often perform read-based profiling of selected genes (or markers) obtained from unassembled shotgun metagenomics reads to classify taxonomy or annotate genes. They then compare the findings with reference databases. Taxonomic binning can make use of similar DNA compositions or nucleotide patterns, such as k-mer lengths, GC content, or gene homology (57). An example of this is the Kraken algorithm, which utilizes unique k-mer distributions in sequences to assign taxonomy (58). On the other hand, MetaPhlAn2 differentiates between different types of microorganisms and calculates their relative abundance by using particular genes that are unique to each group (59).

Despite recent advancements in computational analysis tools and sequencing methods, various factors can still introduce biases and inaccuracies in metagenomics shotgun assembly. Metagenomics shotgun assemblies can employ a combination of *de novo* and reference genome-based approaches, each with its own set of challenges (60).

The Overlap, Layout, Consensus assembly method, commonly used in whole genome sequencing, is not feasible for metagenomic shotgun data due to its high processing demands. Consequently, many new assembly algorithms use the de Bruijn graph approach, such as MEGAHIT (61), MetaVelvet (62), IDBAUD (63), and metaSPADES (64, 65). In reference-guided metagenomic assembly, like MetaCompass (66), contigs are reassembled by aligning sequencing reads to reference databases, but the performance is constrained by the quality of the database and the availability of reference genome sequences (67).

The errors and biases in metagenomic shotgun assembly can be classified into two primary categories: computational challenges and experimental issues. From a statistical perspective, the analysis of microbiome data, including shotgun metagenomics, faces the usual challenges of count data analysis, such as skewed distribution, zero inflation, and over-dispersion (68). Additionally, the experimental process and quality control filtering can result in highly variable and noisy data, which requires normalization to ensure comparability of microbiome abundances among different samples.

The increasing presence of environmental contaminants (ECs) due to human activities has created significant ecological and health challenges. As these pollutants accumulate in ecosystems, they threaten human well-being and various organisms. Recent research has focused on the gut microbiota's role in health, given its influence on metabolism, immunity, and the effects of toxins. Advanced computational tools and artificial intelligence (AI) have become crucial in analysing complex microbiome data, aiding in the identification of disease biomarkers and understanding microbiota interactions. This review examines how these technologies enhance microbiome research, offering insights into biomarker discovery, predictive modelling, and strategies to mitigate the health impacts of ECs (69).

The exponential increase in the number of metagenome-assembled genomes, coupled with advancements in assembly and binning tools, has provided invaluable insights into the presence of previously undescribed organisms and their genetic makeup (70, 71). However, the vast majority of the microbiome diversity remains unexplored, highlighting the need for continued research and development in metagenomics analysis methods.

Despite the rapid advancements in computational tools and sequencing technologies, various factors continue to introduce biases and inaccuracies in metagenomics shotgun assembly. The challenges encompass both computational and experimental issues. From a statistical standpoint, the analysis of microbiome data faces common challenges such as skewed distributions, zero inflation, and over-dispersion. Additionally, the experimental process and quality control measures can result in highly variable and noisy data, requiring normalization to ensure comparability across samples. While the exponential increase in metagenome-assembled genomes has provided valuable insights, the vast majority of microbiome diversity remains unexplored, underscoring the need for ongoing research and development in metagenomics analysis methods (72).

### 4.2 Metabolomics and metaproteomics

This field explores the comprehensive collection of tiny molecules (metabolites) found in a biological system, namely the human stomach. Metabolomics in the field of gut microbiome study is concerned with the identification and quantification of the metabolites generated by both the gut microorganisms and the human host (73). These metabolites are indicative of the metabolic activities of the gut environment and can offer vital information about the functional capabilities of the microbiome. The primary goal of metabolomics analyses is to study the metabolites produced by bacteria and their interactions with the metabolism of both the microbiota and the host (74, 75). These approaches are frequently used to measure small amounts of substances, including as antibiotics, antibiotic metabolites, and products that are produced during the metabolism of bacteria and the host.

Metabolomics and Metaproteomics are crucial techniques in microbiome research that together provide a comprehensive understanding of microbial functionality (76). Metabolomics focuses on analysing the small molecules produced by microbial metabolism, such as amino acids, lipids, and sugars, offering direct insights into the biochemical activities within a microbial community (77). This approach helps reveal active metabolic pathways, detect shifts in microbial processes, and identify biomarkers linked to health or disease. Metaproteomics, on the other hand, examines the proteins actively produced by the microbiome, linking gene expression to protein production and biological functions. By identifying and quantifying these proteins, metaproteomics sheds light on the operational metabolic pathways and microbial responses to environmental stimuli (78). The integration of metabolomics and metaproteomics allows researchers to connect microbial gene expression with functional outcomes, offering a detailed view of the microbiome's role in health, disease, and environmental interactions.

### 4.3 Metatranscriptomics

It involves the comprehensive analysis of the complete set of RNA transcripts present in a microbiome sample at a given time. This technique provides insights into the active gene expression profiles of microbial communities, revealing which genes are expressed and at what levels. By analyzing mRNA, researchers understand which metabolic pathways and biological processes are active in the microbiome, offering a snapshot of the community's functional capabilities. This approach helps elucidate the functional roles of different microbes and their contributions to overall microbiome activity, which is crucial for understanding how microbial communities respond to environmental changes, disease states, or treatments (79).

This subject is centred on the examination of messenger RNA (mRNA) transcripts produced by cells in a given sample. Through the quantification of mRNA levels, we can obtain valuable insights into the genes that are currently undergoing expression by both the gut microorganisms and the host intestinal cells. Metatranscriptomics was first conceived and developed in 2005 as a result of pioneering investigations that sought to identify genes expressed in environmental samples (80, 81).

Metatranscriptomics and metabolomics are complementary approaches in microbiome research, each providing unique insights into the functional dynamics of microbial communities. Metatranscriptomics examines RNA transcripts to identify which genes are actively expressed under various conditions, offering a real-time view of microbial activities and their responses to environmental changes, stressors, or host interactions. Techniques like RNA-Seq are commonly used to capture a comprehensive snapshot of the transcriptome, enabling functional profiling that identifies active metabolic pathways and regulatory networks (82). This is particularly valuable in understanding how microbes adapt to different environmental and host-associated contexts, though the complexity and variability of microbial communities pose challenges in interpreting gene expression data.

## 5 Technological platforms in multi-omics techniques

### 5.1 Technological platforms in metaproteomics and metabolomics

Metaproteomics and metabolomics are burgeoning scientific disciplines that have made substantial strides in the investigation of the microbiome. The production of metabolomics data differs significantly from that of metatranscriptomics and metagenomics, as the latter two rely heavily on sequencing. Metabolites are typically detected and measured by employing a mix of chromatography techniques, such as gas chromatography and liquid chromatography, together with detection methods like nuclear magnetic resonance and mass spectrometry. NMR provides consistent, quantitative precision, and unambiguous, definitive results for non-destructive, complex structure determination. NMR can target different atom nuclei, such as hydrogen (1H-NMR), carbon (13C-NMR), and phosphorus (31P-NMR), offering further information on specific metabolite types. The utilization of LC-NMR greatly enhances the advantages of NMR-based metabolomics, effectively reducing the complexity of samples. Each analytical platform has its own advantages and disadvantages, and the choice of the platform depends on the focus of the study, the nature of the samples, cost, accessibility, and available expertise. Metabolomics contains the downstream products of genomic, transcriptomic, and proteomic processes, and the metabolome is sensitive to various genetic and environmental stimuli, requiring careful experimental design to reduce confounders and optimize information recovery (83–87).

Metaproteomics and metabolomics are complementary approaches that provide deep insights into the functional activities of microbial communities. Metaproteomics focuses on identifying and analysing the proteins actively produced by microorganisms, offering a real-time view of their metabolic and regulatory pathways. This method is particularly valuable in complex ecosystems like soil, oceans, and the human gut, where it helps uncover microbial interactions and responses to environmental changes. Metabolomics, on the other hand, studies the small molecules or metabolites produced within these communities, giving a detailed picture of the biochemical processes at play. By linking specific proteins identified through metaproteomics to their corresponding metabolic outputs revealed by metabolomics, researchers can achieve a comprehensive understanding of microbial functions. This integrated approach is especially useful in health-related research, shedding light on the role of the microbiome in conditions like obesity, diabetes, and inflammatory diseases. As these fields continue to advance, the combined use of metaproteomics and metabolomics will be crucial for developing microbiome-based therapies, improving environmental management practices, and driving innovations in biotechnology and personalized medicine.

### 5.2 Technological platforms in metatranscriptomics

Metatranscriptomic approaches, which collect the RNA transcribed by microbial cells, utilize similar analytical principles as shotgun metagenomics, elucidating the active functional profile of a microbial community through the analysis of all population-expressed genes. A snapshot of gene expression, the metatranscriptome captures the complete mRNA in a given sample at a precise instant and under specific conditions. Shotgun metagenomics and metatranscriptomic techniques mostly rely on Illumina sequencing methods, with the HiSeq or NovaSeq (88) instrument families being the most commonly used due to their cost-effectiveness per base and ability to process large amounts of data. On the other hand, there has been a shift towards using PacBio and Oxford Nanopore sequencing technologies (89) to take advantage of their longer read lengths, which make it easier to map the genetic information of a reference genome and identify genes (17, 90). The usual method for sequencing the microbiome sample involves isolating total RNA, enriching RNA, fragmenting it, synthesizing cDNA, and producing transcriptome libraries.

Until recently, these techniques were limited to a relatively particular assortment of alleles. At this time, shotgun sequencing of complete metatranscriptomics is feasible using metagenomics, and a thorough examination of gene expression across the entirety of the genome provides an elaborate synopsis of the functional attributes and expression patterns of a microbiome. Most of these techniques follow the initial read mapping-based strategy, with de-novo assembly of reads into transcript contigs and supercontigs or mapping of reads to a reference genome constituting a standard metatranscriptomics analysis pathway. Comparable to alignment-based methods in whole-genome sequencing, the initial strategy consists of mapping sequences to reference databases in order to obtain information that can be used to (91–93).

### 5.3 Metagenomics

Metagenomics has previously been used to evaluate the microbial community within a sample or environment, for example, interrogating the gut microbiome and its association with chronic diseases. Metagenomics is progressively being applied as a novel infectious disease diagnostic assay, with two main approaches: shotgun metagenomics, which attempts to sequence the entire genetic content present in a sample, and targeted-amplicon sequencing, which represents a more biased approach to a particular group of microorganisms (37).

Advances in non-targeted short-read sequencing made during the Human Genome Project, particularly innovations by J. Craig Venter and his team, gave rise to shotgun sequencing, wherein nucleic acid from a sample is fragmented and the entire population of fragments is subjected to unbiased sequencing followed by characterization and assignment of the sequenced fragment. This method serves as a census of organisms in the original sample.

## 6 Functional classifications in microbiome research

### 6.1 Metagenomics: leveraging computational tools for microbial insights

Researchers possess the capacity to identify discrepancies in metabolic activity across unique microbial populations, as well as to scrutinize the taxonomic makeup of a microbiome (16). Through the implementation of software applications such as PICRUSt or Tax4Fun (73), it is feasible to predict a functional profile by utilizing 16S sequencing data. By utilizing the relative abundance of taxa in the community and the reference genome for each taxonomic present, these programs are capable of predicting the likely functionality of genes. However, it is important to note that these methods only offer an approximation, as they neglect to consider the true expression levels of proteins and rely significantly on reference genomes and their annotations.

Metatranscriptome and shotgun techniques both facilitate functional analysis. Gene predictions are produced subsequent to the compilation of a metagenome through the utilization of software tools such as Glimmer-MG (94) and MetaGeneMark (95). Functional annotation is executed subsequent to the identification of coding genes through the implementation of computationally intensive searches predicated on protein sequence homology. Typically, databases of orthologues (e.g., EggNOG or COG), and enzymes, or protein domains and families are queried using UBLAST and USEARCH-based queries (96). Software applications such as Pathfinder can be employed to perform pathway enrichment analysis, classification, and scoring purposes. KEGGscape (97) and similar applications may be utilized in a similar fashion to construct a metabolic network (98, 99).

A multitude of publicly accessible automated algorithms has been devised to manage the substantial computational demands and tool sets associated with various tasks, including but not limited to quality filtering, gene calling, functional annotation, and fundamental statistics and visualization using MG-RAST and MEGAN-CE (100). While these approaches have significantly advanced our understanding of microbial communities, it is important to note that culturing of taxa is still essential to determine the ecological significance of function (101). Cheaper sequencing has democratized the application of metagenomics, but has also come at the cost of reduced sequence length, resulting in poor gene annotation and overestimates of bacterial richness and abundance. Recent improvements in sequencing technology are beginning to provide reads of sufficient length for accurate annotation and assembly of whole operons and beyond, that will once again enable experimental testing of gene function and re-capture the early successes of metagenomics investigations (98).

As sequencing projects remain largely biased towards genomes linked to human interests, some serious initiatives are being launched for sequencing organisms that represent all branches of the tree of life. Concomitant with the genomic revolution, unprecedented advances in sequencing technology have also led to the emergence of the field of metagenomics, which offers a novel, revolutionary approach for studying life in different environments (102).

This paper has provided an overview of the current state of functional classifications in metagenomics, highlighting the computational tools and methods available for researchers to gain insights into microbial communities.

### 6.2 Functional profiling in metatranscriptomics

Metatranscriptomics has emerged as a powerful tool in unravelling the intricate dynamics of the gut microbiome, shedding light on the ongoing biological processes and metabolic pathways that shape this complex ecosystem (103). Through the analysis of RNA sequence data, researchers can categorize and characterize the genes that are actively expressed, providing insights into the functional behaviours of the resident microbial communities (104).

The process typically involves aligning the metatranscriptomic data obtained from microbiome samples to specific pathways and genomes, such as the Kyoto Encyclopedia of Genes and Genomes (105). Bioinformatics tools like SOAPdenovo have been employed to construct and align these metatranscriptomic datasets (106). Comparative analyses across different health and disease states allow researchers to identify the pathways that experience increased or decreased activity in response to various factors.

The subsequent annotation of these results using databases like Gene Ontology, Clusters of Orthologous Groups, and Swiss-Prot enables a more comprehensive understanding of the metabolic and functional capabilities of the gut microbiome (107).

The applications of functional profiling in metatranscriptomics extend beyond the mere cataloguing of microbial gene expression. Techniques like stable isotope probing have been utilized to isolate the transcriptomes of specific aerobic bacteria found in environmental samples, significantly advancing the field of metabolomics by enabling the targeted investigation of key microbial species (19).

Functional Profiling in Metatranscriptomics uses RNA sequencing (RNA-seq) to capture gene expression across microbial communities, offering insights into their real-time biochemical activities. Unlike genomics, which identifies potential genetic capabilities, metatranscriptomics reveals the actual molecular processes occurring in microbes within their natural environments. This makes metatranscriptomics essential for understanding microbial behaviour, host-microbe interactions, and identifying biomarkers. Despite challenges like RNA degradation and the need for extensive sequencing, Metatranscriptomics holds significant potential for advancing our knowledge of microbial ecosystems, with broad implications for health, biotechnology, and environmental management (108).

Overall, the integration of metatranscriptomics with functional profiling has opened new avenues for understanding the complex interplay between the gut microbiome and host health, paving the way for more targeted and personalized therapeutic interventions (26, 27, 37, 98).

## 7 Key bioinformatics tools for phylogenetic and microbiome analysis

### 7.1 Phenotypic classification

Phylogenetic and microbiome analyses are essential for exploring microbial diversity, structure, and function. Phylogenetic tools elucidate evolutionary relationships and taxonomic classifications of microorganisms. Microbiome analysis tools, including those for 16S rRNA gene sequencing, identify microbial taxa and their functional potential. Multi-omics approaches integrate genomic, transcriptomic, proteomic, and metabolomics data to offer a comprehensive view of microbial interactions. These bioinformatics tools are crucial for advancing our understanding of microbial ecology, health impacts, and therapeutic possibilities (109).

Phyloseq: Phyloseq is an R package designed for the analysis and visualization of microbiome data. It provides tools for importing, analysing, and graphically displaying phylogenetic trees, taxonomic composition, and diversity metrics. Phyloseq integrates well with other R packages, making it a flexible choice for custom analyses and visualizations (110).

USEARCH is a comprehensive software package used for clustering and analysing 16S rRNA gene sequences. It supports a wide range of functionalities, including *de novo* OTU clustering, chimera detection, and sequence alignment. USEARCH is highly efficient, capable of processing large datasets quickly, which is crucial for handling the vast amounts of data generated in microbiome studies. Although it is a commercial tool, its performance and speed in OTU clustering make it a preferred choice for researchers seeking high throughput and accuracy in sequence analysis (111).

BEAST (Bayesian Evolutionary Analysis Sampling Trees): BEAST is a powerful tool used for Bayesian analysis of molecular sequences. It is particularly well-suited for phylogenetic analysis involving time-stamped sequences, allowing researchers to infer phylogenies and estimate divergence times. BEAST is commonly used in evolutionary biology and can be applied to microbiome studies to explore the evolutionary history of microbial taxa (112).

FastTree: FastTree is an efficient tool for constructing approximately-maximum-likelihood phylogenetic trees from large alignments. It is widely used in microbiome studies for its ability to handle large datasets quickly, making it ideal for constructing phylogenetic trees from 16S rRNA gene sequences or other marker genes (113).

RAxML (Randomized Axelerated Maximum Likelihood): RAxML is a popular software tool for maximum-likelihood-based phylogenetic inference. It is used to create phylogenetic trees based on nucleotide or amino acid sequences and is known for its speed and accuracy, making it a valuable tool for large-scale microbiome phylogenetic analyses (114).

MEGA (Molecular Evolutionary Genetics Analysis): MEGA is a comprehensive software suite for conducting a variety of phylogenetic and statistical analyses. It allows users to build phylogenetic trees, estimate evolutionary distances, and conduct hypothesis testing. MEGA is user-friendly and widely used in evolutionary studies, including those involving microbial communities (115).

### 7.2 Evaluation of microbiome diversity metrics: alpha and beta diversity

The assessment of microbiome variations often involves the comparison of alpha and beta diversity measurements, either individually or in combination (116). Alpha diversity metrics evaluate the level of diversity present within a given sample, enabling comparisons across different groups. For instance, it is common to compare the mean species diversity of samples collected from a cohort of organisms afflicted with a particular ailment to that of a cohort devoid of the ailment (117). Species richness estimators, such as observed OTUs and the Chao1 index, are frequently utilized alpha diversity metrics. Additionally, the Shannon and Inverse Simpson indices are employed to evaluate both species richness and evenness. An alternative approach to quantifying diversity is the utilization of phylogenetic richness estimators, like Faith's phylogenetic diversity (117). Richness and evenness estimators, including Shannon and Inverse Simpson, are regarded as more robust due to their reduced sensitivity to sample sequence count variability. The Shannon index is predominantly impacted by the existence of rare operational taxonomic units, while the Inverse Simpson index is predominantly impacted by the presence of numerous or dominant OTUs (10).

As a diversity metric, beta diversity evaluates the dissimilarity of sample characteristics. The distance matrix is frequently obtained by computing the distance between every pair of samples, which is a common method of deriving it. The Bray-Curtis dissimilarity is a widely employed technique for computing beta diversity. It is a quantitative metric that compares two communities by considering the abundance of various taxa. The Weighted Unifrac distance is a metric that measures dissimilarities between two communities by considering phylogenetic relatedness alongside taxonomic abundances. In contrast, the unweighted Unifrac distance is a qualitative measure that solely considers the existence or absence of taxa (117, 118).

### 7.3 Visualization and statistical techniques in metagenomics research

Metagenomics research, which involves the study of the collective genetic material of microorganisms within a given environment, has experienced a surge in popularity in recent years. One of the key aspects of such investigations is the application of visualization and statistical methods to analyse and interpret the complex datasets generated.

Microbiome studies often entail the comparison of specific taxa, functional elements, microbial diversity, and control group characteristics between different groups. However, the inherent complexity of these datasets, including high dimensionality and potential zero-inflation, presents challenges when employing standard statistical methods. To address these issues, researchers have developed and refined various visualization and statistical techniques tailored for metagenomics data.

Visual inspection of the data is a common starting point, as it can reveal potential correlations or patterns that may warrant further investigation using more rigorous statistical approaches. Dimension-reduction techniques, such as principal coordinate analysis and principal component analysis, are frequently utilized to convert distance matrices into two- or three-dimensional graphical representations of sample relationships. These visualizations allow for the classification and annotation of samples based on relevant metadata (119) (Thomas et al., 2012). Visual comparison of multiple metagenomes and statistical comparison of two metagenomes at a time have been implemented in tools like MEGAN. These methods provide a means to effectively explore and compare large metagenomics datasets (119). As the field of metagenomics continues to evolve, the development and refinement of computational approaches for data analysis and integration remains an active area of research (119).

The transition from classical microbiology to modern metagenomics has been facilitated by advancements in high-throughput DNA sequencing technologies, which have enabled the direct genetic analysis of complex microbial communities (16). The vast amounts of data generated by these technologies require the integration of various computational methods to collect, process, and extract meaningful biological insights.

#### 7.3.1 Utilizing ordination methods for enhanced visualization and analysis in metagenomics

Ordination techniques are essential tools in metagenomics research for visualizing and interpreting complex microbial community data. These methods help researchers to reduce the dimensionality of large datasets, identify patterns, and explore relationships between microbial communities and environmental factors. Below are some key ordination techniques:

Principal Component Analysis (PCA) is a technique used in metagenomics to reduce data dimensionality by transforming complex datasets into principal components. This method simplifies data visualization by focusing on the principal axes that capture the most variability, which helps in identifying patterns such as clusters, outliers, and trends across samples. PCA results are typically displayed in scatter plots of the first few principal components (120). In contrast, Non-metric Multidimensional Scaling (NMDS) is designed to preserve the rank order of distances between samples rather than their exact numerical distances. This method is particularly suited for non-parametric data and is used to explore beta diversity by visualizing how microbial communities differ across samples. NMDS provides insights into sample dissimilarities by representing them in two or three dimensions, revealing gradients or clusters without relying on parametric assumptions (121).

Canonical Correspondence Analysis (CCA), on the other hand, integrates aspects of both principal component and regression analyses to investigate how environmental variables influence microbial community composition. CCA correlates community changes with factors like pH or nutrient levels, offering a way to understand ecological drivers of microbial diversity. The results are often presented in biplots, where microbial taxa and environmental variables are depicted together, highlighting their associations and providing insights into the ecological dynamics of the communities (122).

### 7.4 Statistical techniques in metagenomics research

Statistical techniques are essential for analysing and interpreting metagenomics data, providing insights into microbial community differences, sample classification, and biomarker discovery (123). PERMANOVA (Permutational Multivariate Analysis of Variance) is a non-parametric method that tests the significance of differences in microbial community composition across various groups or conditions without assuming a specific data distribution (124). It evaluates whether observed differences among groups exceed what would be expected by chance, with results typically presented in distance-based dissimilarity matrices and permutation test outputs. Random Forests is a machine learning technique that builds multiple decision trees to classify samples or predict outcomes based on microbial compositions. It is robust to overfitting and can handle large datasets, with results visualized through feature importance plots and confusion matrices to assess classification performance. LEfSe (Linear Discriminant Analysis Effect Size) combines linear discriminant analysis with effect size measurements to identify biomarkers by highlighting features that significantly differ between groups (125). This method is used to discover microbial taxa or functional genes that are discriminatory between conditions, with results often displayed in bar plots or cladograms to illustrate the magnitude of differences and key drivers of microbial community variations.

### 7.5 Differential abundance analysis

Differential abundance analysis in metagenomics identifies and visualizes changes in microbial taxa between conditions. It helps understand how microbial communities respond to factors like environmental changes, dietary interventions, or diseases. Two key visualization techniques are volcano plots and MA plots.

Volcano Plots display statistical significance vs. fold change, with the *x*-axis showing the magnitude of change in abundance and the *y*-axis representing significance [usually -log10 (*p*-value)]. This plot highlights taxa with substantial changes and significant differences, facilitating the identification of key drivers in microbial shifts (126).

MA Plots visualize the relationship between mean abundance and fold change. The *x*-axis represents average abundance, while the *y*-axis shows log-fold change between conditions. This plot reveals how changes in abundance correlate with overall levels and helps identify trends or biases (127).

## 8 Network analysis and machine learning: connecting the dots

These “omic” data kinds each contribute to the puzzle. However, multi-omics’ true potential lies in its ability to integrate these diverse information. Advanced computational tools and network analysis methods find information layer connections and interactions. We can link chemicals to their microbial creators by combining metabolomics and metagenomics data. Integrating transcriptomic and metabolomics data can reveal how gut bacteria metabolic activities affect host intestinal cell gene expression. Microbiome community connections are often studied using network analysis. In various contexts, correlation networks show community structure disruptions. They can also study interkingdom relationships between environmental elements, metabolites, clinical features, and other bacteria in the microbial community. Graphical networks often compare interactions in ill vs. healthy states or show species co-occurrence or mutual exclusion. Machine learning methods can manage complex data and identify useful properties in microbiome datasets with many attributes, making them appealing for evaluation (128). Machine learning aids in deciphering the intricate relationships within the gut ecology and transforming the varied biological data into useful insights (129).

This method employs various types of networks to reveal complex relationships between microbial taxa or genes, offering insights into community structure, functional relationships, and ecological dynamics. Two primary types of networks used in this analysis are co-occurrence networks and correlation networks.

Co-occurrence Networks are designed to illustrate interactions between microbial taxa within a community. In these networks, nodes represent different taxa, and edges denote the correlations or interactions observed between them. Positive edges suggest that taxa frequently occur together, while negative edges indicate that their presence might be mutually exclusive. By visualizing these relationships, researchers can identify groups of taxa that may influence each other's abundance or activity. For example, co-occurrence networks can help uncover ecological partnerships or competitive interactions, providing insights into the stability and functionality of microbial communities (130).

Correlation Networks focus on identifying potential interactions or co-occurrences between microbial species or genes based on their correlation patterns. In these networks, nodes represent microbial species or genes, and edges represent significant correlations between them. Correlation networks are valuable for exploring how different microbial entities relate to each other in terms of abundance or gene expression. They can reveal patterns of co-expression or co-occurrence that might suggest functional relationships or shared environmental preferences. For instance, identifying highly correlated species can provide clues about cooperative metabolic pathways or shared responses to environmental changes (131).

In the area of gut microbiome research, the integration of network analysis and machine learning (ML) has been greatly enhanced by the development of various computational tools. These tools are designed to analyse complex datasets, identify patterns, and map interactions within microbial communities (132).

In microbiome research, tools for unsupervised learning, such as PCA (Principal Components Analysis) and PCoA (Principal Coordinate Analysis), are essential for simplifying high-dimensional data and revealing the structure of microbial communities (133). These techniques reduce the complexity of large datasets by projecting them onto fewer dimensions while preserving critical information. This approach is vital in studies where datasets may contain vast numbers of microbial species or genes. For instance, a few tools such as

QIIME II, a specialized platform for microbiome analysis, incorporates PCA and PCoA tools to visualize variations in microbial composition across different samples, such as comparing gut microbiota between healthy individuals and those with diseases. This platform enables researchers to track microbial community structures over time and under different conditions, offering valuable insights into the factors influencing microbial diversity (25).

Another widely used tool is Emperor, an interactive visualization software that works seamlessly with outputs from QIIME 2 and other microbiome analysis pipelines. Emperor allows researchers to explore and interpret the results of PCA and PCoA in a dynamic, three-dimensional environment, facilitating the identification of patterns and relationships within microbial communities (134). Using the application of these tools, researchers can acquire a deeper understanding of the ways in which microbial communities assemble or separate, ultimately assisting in the recognition of significant microbial taxa and advancing the creation of targeted measures or treatments. These unsupervised learning tools are crucial for transforming complex, high-dimensional data into interpretable visualizations, driving forward the field of microbiome research (135).

## 9 Key functional annotation tools for microbial genomics

Key functional annotation tools for microbial genomics, such as KEGG, COG, EggNOG, and Pfam, provide critical insights into metabolic pathways, protein functions, and evolutionary relationships, enabling comprehensive analysis of microbial genomes and their functional roles (Table 2).

**Table 2:** Functional classification and annotation tools used in Gut microbiome analysis.

Tool/database	Purpose	Description	Examples of use	Outputs	References
KEGG (Kyoto Encyclopedia of Genes and Genomes)	Pathway mapping and functional annotation of genes	Provides pathway-based annotations and enzyme codes	Metabolic pathway analysis, functional gene annotation	Pathway maps, enzyme roles	(136)
COG (Clusters of Orthologous Groups)	Classification of proteins into orthologous groups	Groups proteins with similar functions from different organisms	Functional prediction, phylogenomic studies	Functional categories, evolutionary history	(137)
EggNOG (Evolutionary Genealogy of Genes: Non-supervised Orthologous Groups)	Functional annotation and evolutionary classification	Non-supervised clustering of proteins into orthologous groups	Annotating metagenomic data, evolutionary studies	Functional roles, orthologous relationships	(138)
Pfam (Protein Families)	Identifying protein families and functional domains	Database of protein families represented by multiple sequence alignments	Predicting protein function, detecting conserved domains	Protein domains, functional annotations	(139)

KEGG offers valuable insights into metabolic pathways and enzyme functions, essential for understanding the biochemical processes within microbial communities. It provides detailed pathway maps and enzyme roles that facilitate the analysis of microbial metabolism and functional capacities (24). COG classifies proteins into orthologous groups, enabling researchers to predict protein functions based on evolutionary conservation across different organisms. This classification supports functional prediction and elucidates the evolutionary history of microbial proteins (140). EggNOG complements COG by employing non-supervised clustering methods to group proteins into orthologous categories, aiding in the annotation of metagenomic data and evolutionary studies. It provides crucial information on functional roles and orthologous relationships, which are vital for interpreting the functional potential of microbial genomes (141). Pfam focuses on identifying protein families and functional domains through multiple sequence alignments, crucial for predicting protein functions and detecting conserved domains, thus enhancing our understanding of protein functionality and evolutionary conservation in microbial species (142).

## 10 Inferring microbial interaction networks from microbiome data: a comparative analysis of SparCC, CCLasso, and SPEIC-EASI

The study of microbial communities and their interactions within complex ecosystems, such as the human gut, has become a crucial area of research in the field of microbiome science. Researchers have developed several computational methods to infer these intricate networks of microbial interactions from high-throughput sequencing data.

One common approach is to examine pairwise associations between microbial taxa, where networks are established by measuring the similarity or correlation coefficients between pairs of variables. Three commonly used software programs for this purpose are SparCC, CCLasso, and SPEIC-EASI (143, 144).

SparCC and CCLasso are two popular methods that account for the inherent compositional nature of microbiome data, which can lead to spurious correlations if not properly addressed. However, the SPEIC-EASI model is currently the most commonly utilized approach due to its strong ability to estimate interactions using techniques for both sparse neighbourhood and inverse covariance selection, following the initial CLR transformation of the count data (144).

Regression-based techniques, such as sparse regression, Dirichlet-multinomial regression, and generalized boosted linear models, can also be employed to forecast the abundance of a particular species based on the abundance of various combinations of other species (145). Another strategy relies on the presence-absence patterns of taxa in relation to distinct phenotypes or outcomes, which is often referred to as association rule mining.

These complex network analyses, together with machine-learning approaches, provide a more comprehensive understanding of the intricate relationships within the gut ecosystem. They enable researchers to not only identify the microbial players, but also understand their functional roles and how they interact with each other and with the human host.

## 11 Comparing the interpretability and performance of machine-learning, random forest and decision tree models in clinical predictive modelling

Machine learning is a field within artificial intelligence that enables independent knowledge acquisition and operational improvement through the utilization of input data, without the need for explicit programming (146). Random Forest, an ensemble machine learning technique, is commonly employed for classification and regression tasks. It is often utilized to uncover significant taxonomic and clinical variables that can differentiate various phenotypes or classifications, or predict specific outcomes (147).

Despite being less precise and reliable, techniques like CART analyses are more interpretable and, consequently, more therapeutically actionable. Decision trees allow researchers to gain insights into the significance of variables, their cutoff points, and their order of importance. It is crucial to note that these models should always undergo cross-validation, either through sample and replacement, or by using independent cohorts for training, testing, and validation (148).

The goal of this comparative study is to assess the effectiveness of decision trees, such as those used in CART analyses, and Random Forest models in the analysis of biomedical data. Specifically, we aim to evaluate the trade-offs between model interpretability and predictive performance, as these factors are crucial considerations for clinical decision-making.

Random Forest is an ensemble machine learning technique that constructs multiple decision trees and aggregates their predictions to improve the overall accuracy and stability of the model. This method has been shown to be effective in uncovering significant variables that can differentiate various phenotypes or classifications, or predict specific outcomes in a wide range of scientific fields (149).

On the other hand, decision tree-based models, such as CART, are more interpretable, enabling researchers to gain insights into the significance of variables, their cutoff points, and their order of importance. These insights can be more readily translated into actionable clinical decisions (150).

### 11.1 Unsupervised and supervised machine-learning in gut microbiome analysis

Machine learning algorithms can be broadly classified as either unsupervised learning or supervised learning, and they have been extensively applied in the study of intestinal microbiota (Table 3). Unsupervised learning methodologies obtain and classify novel hidden patterns uniquely from given datasets in which the dependent variables are unknown, and they are frequently described as predictions that are driven by data (160). Dimension reduction and clustering analysis are two main categories of unsupervised learning techniques. For the visualization of omics data, principal components analysis, principal coordinate analysis, and t-distributed stochastic neighbour embedding (t-SNE) (156) are typical dimension reduction techniques that extract a subset of crucial variables from the high-dimensional feature space. Clustering techniques, such as hierarchical clustering, k-means clustering, and self-organizing map, are frequently employed to partition a collection of entities into multiple clusters according to their similarities or dissimilarities. In the study of intestinal microbiota, clustering analysis has been used to discern novel patterns, such as the identification of co-abundance gene groups and enterotypes of the human microbiota.

**Table 3:** Machine learning techniques for microbiome data.

Category	Technique/algorithm	Application	References
Supervised learning	Random forest	Classification and regression to identify significant taxonomic and clinical variables.	(151)
	CART analysis	More interpretable decision trees for clinical applicability.	(152)
	K-Nearest Neighbor (KNN)	Classification of samples based on nearest neighbors in the feature space.	(153)
	Support Vector Machine (SVM)	Used for classification and regression problems.	(154)
	Naive Bayes (NB)	Probabilistic classification based on Bayes’ theorem.	(129)
	Random Forest (RF), LightGBM, XGBoost	Ensemble methods for strong performance and interpretability.	(152)
Unsupervised learning	Principal Components Analysis (PCA)	Dimension reduction technique for visualizing high-dimensional omics data.	(155)
	t-Distributed Stochastic Neighbor Embedding (t-SNE)	Visualization of complex data in lower dimensions.	(156)
	Hierarchical Clustering	Groups samples based on similarity measures, useful for identifying co-abundance gene groups.	(157)
	K-Means Clustering	Partitions samples into clusters based on feature similarity.	(158)
	Self-Organizing Map (SOM)	Visual representation of complex data structures for pattern recognition.	(159)

Unsupervised machine learning algorithms not only derive insights directly from the data and group the data, but also use these insights for data-driven decision making. Supervised learning, on the other hand, utilizes labelled data to train a model that can then make predictions on new, unseen data.

### 11.2 Supervised and supervised machine-learning in gut microbiome analysis

In contrast, supervised learning methods obtain information and infer a function from input data that includes dependent variables for all samples and independent variables (also known as features). Supervised learning is the process of using known dependent variables from a training dataset to create a machine learning model that can predict the outcomes of new samples. ML models can be used to perform classification problems when the dependent variables are categorical. Because the dependent variables are continuous, they can also be used for regression problems.

The application of both unsupervised and supervised learning techniques has been crucial in the study of gut microbiome, enabling researchers to uncover novel patterns, identify enterotypes, and develop predictive models for various health and disease states (118, 161).

## 12 Challenges in current applications

The gut microbiota, a complex and dynamic ecosystem of trillions of microorganisms, plays a pivotal role in human health and disease. Understanding the intricate interactions within this microbial community and their impact on the host requires advanced computational tools and cutting-edge machine learning (ML) approaches. These technologies have revolutionized the field of microbiome research, enabling the identification of novel biomarkers for disease diagnosis, treatment, and personalized medicine (162).

One of the primary challenges in gut microbiota research is the vast complexity and diversity of microbial communities. The gut microbiome consists of a host of bacterial, viral, fungal, and archaeal species, each contributing to the overall ecosystem. Traditional sequencing methods, such as 16S rRNA gene sequencing, provide a restricted view of microbial diversity, often missing rare or less abundant species (163). To overcome this, advanced computational tools like metagenomics and metatranscriptomics have been developed to capture a more comprehensive concept of microbial diversity and function. However, these approaches generate massive amounts of data, creating a need for sophisticated data processing and analysis pipelines (164).

Machine learning has emerged as a powerful approach to handle the complexity of microbiome data. By leveraging algorithms capable of detecting patterns and making predictions based on large datasets, ML can identify key microbial features associated with specific health outcomes (165). In case, ML models can be trained to distinguish between healthy and diseased microbiomes, potentially leading to the discovery of biomarkers for conditions like inflammatory bowel disease (IBD), obesity, and colorectal cancer. Despite these advancements, several challenges persist, including the need for large, well-annotated datasets and the risk of overfitting models to specific cohorts, which can limit the generalizability of findings (129).

Another significant challenge in the application of ML to gut microbiota research is the integration of multi-omics data. The gut microbiome interacts with various host systems, influencing metabolic, immune, and neural processes. To capture this complexity, researchers are increasingly turning to multi-omics approaches, integrating data from genomics, transcriptomics, proteomics, metabolomics, and more. Tools like Multi-Omics Factor Analysis (MOFA) and Integrative Genomics Viewer (IGV) have been developed to facilitate this integration, enabling a more holistic understanding of microbiome-host interactions. However, the integration of such diverse data types requires advanced computational frameworks and careful consideration of data harmonization and normalization techniques (166).

Artificial intelligence (AI) and machine learning (ML) in microbiome analysis face significant challenges, particularly regarding reproducibility, replicability, robustness, and generalizability. These challenges are exacerbated by the complex, interdisciplinary nature of microbiome research, where slight variations in methods, data processing, and analytical frameworks can lead to divergent results (152). Reproducibility issues are not merely technical but are deeply rooted in the lack of standardized methods, the complexity of biological data, and the evolving nature of computational tools. The historical example of Antonie van Leeuwenhoek's struggle to have his microbial observations accepted illustrates the enduring difficulty of ensuring that scientific work can be precisely replicated by others. Modern examples, like the challenge posed by Philip Bourne's group to reproduce their computational analysis, underscore the immense effort required to replicate complex bioinformatics research, even when transparency is prioritized. These challenges highlight the need for greater methodological transparency, standardization, and collaborative efforts to enhance the robustness and generalizability of AI and ML approaches in microbiome research (167).

Furthermore, ethical and privacy concerns associated with microbiome research pose additional challenges. As the field moves towards personalized medicine, where individual microbiome profiles guide treatment decisions, issues related to data privacy, consent, and ownership become increasingly important. Researchers must navigate these challenges carefully, ensuring that the benefits of microbiome-based interventions are realized without compromising patient privacy or autonomy (168).

**Figure 3:**
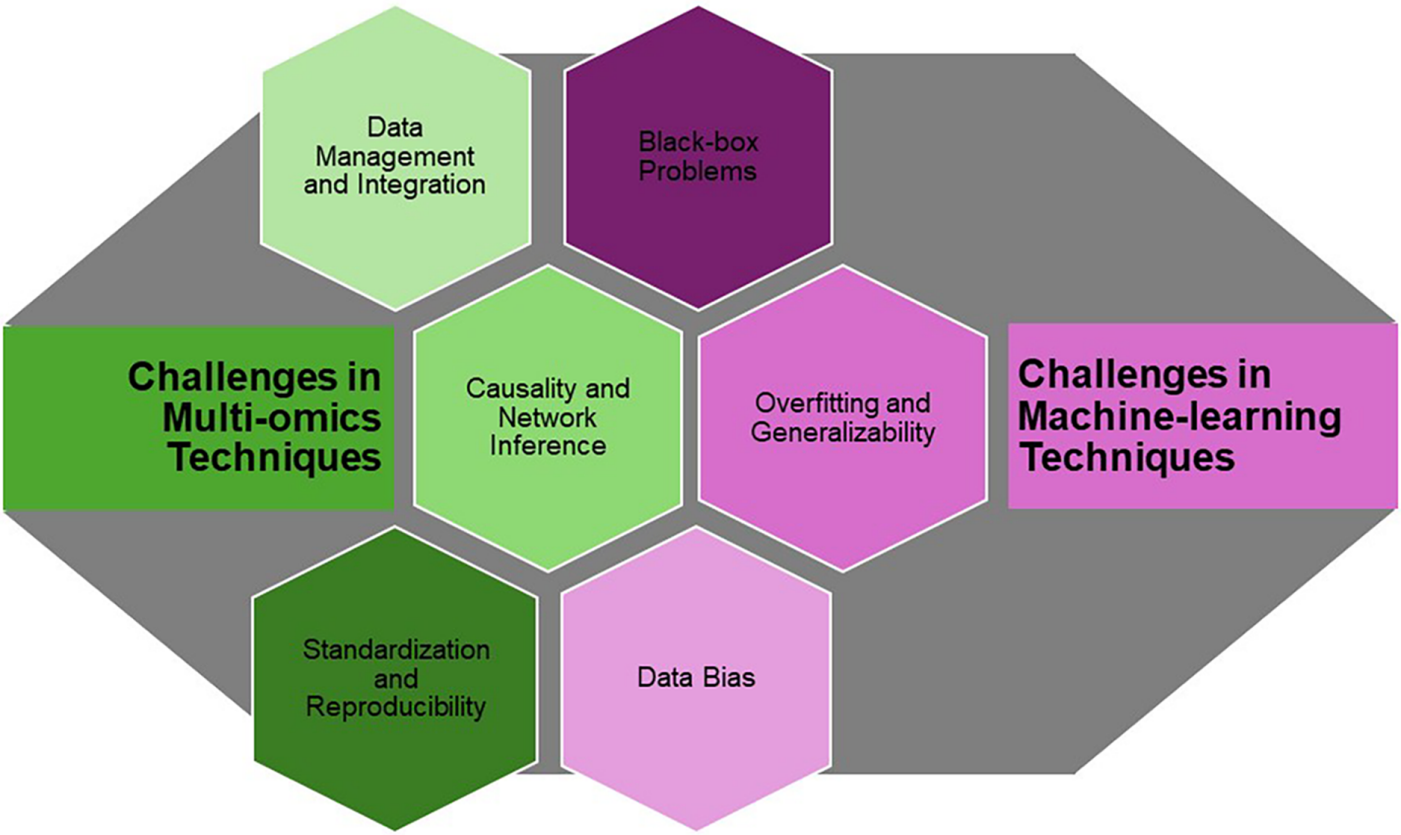
Potential challenges and considerations in multi-omics and machine learning for advancing gut microbiome research.

Despite these challenges, the potential of advanced computational tools and ML in gut microbiota research is immense (Figure 3). The integration of these approaches holds promise for the discovery of novel biomarkers that can revolutionize disease diagnosis and treatment. For instance, biomarkers identified through ML could lead to the development of non-invasive diagnostic tests for gastrointestinal disorders or the creation of personalized probiotics tailored to an individual's unique microbiome composition.

To fully realize these opportunities, ongoing collaboration between microbiologists, data scientists, clinicians, and ethicists is essential. Interdisciplinary teams are needed to develop and refine computational tools, address the challenges of data integration and interpretability, and ensure that microbiome research is conducted ethically and responsibly. Additionally, the development of standardized protocols for data collection, analysis, and reporting will be crucial in advancing the field and enabling the translation of research findings into clinical practice.

## 13 Conclusion

The analysis of the gut microbiome has undergone a transformative shift, driven by the integration of advanced computational tools, multi-omics techniques, and the power of artificial intelligence and machine learning (169). This convergence of technologies is enabling researchers to gain unprecedented insights into the complex dynamics and interactions within the gut microbial ecosystem.

One of the key elements fuelling this progress is the availability of high-throughput sequencing technologies that generate vast datasets across various omics levels, including genomics, transcriptomic, proteomics, and metabolomics. These data-rich studies are in turn supported by a suite of powerful bioinformatics software, such as QIIME and Mothur, which facilitate the processing and analysis of the microbiome data (26, 170).

**Figure 4:**
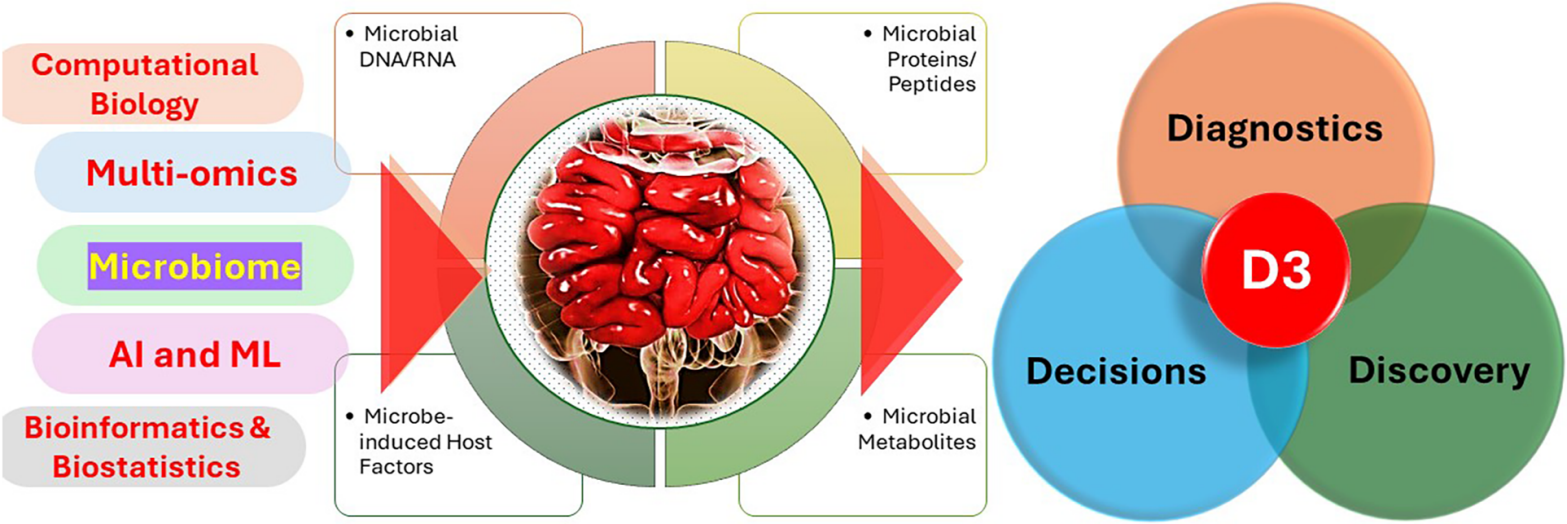
Different computational, bioinformatics, AI & ML approaches in gut microbiome analysis and biomarker discovery for achieving D3 (diagnostics, discovery and decision) and goals of precision medicine.

The multi-omics approach, which combines data from different biological layers, provides a more comprehensive understanding of the gut microbiome. By integrating genomic, transcriptomic, proteomic, and metabolomics information, researchers can uncover intricate relationships between microbial composition, gene expression, protein function, and metabolic profiles. Artificial intelligence and machine learning play a pivotal role in the interpretation of this complex multi-omics data. These computational techniques excel at pattern recognition, enabling the identification of correlations and associations within large datasets. Furthermore, AI and ML models can be leveraged for predictive modelling, helping to forecast disease outcomes or responses to treatments based on microbiome profiles. This, in turn, has led to advancements in disease diagnosis, prognosis, and the development of personalized medicine approaches. The integration of these cutting-edge tools and techniques has opened up new frontiers in gut microbiome research. Researchers can now explore the intricate interactions between the microbiome and the host, uncover biomarkers for disease, and develop more targeted and effective therapeutic interventions (Figure 4). As this field continues to evolve, we can expect to see even more transformative breakthroughs in our understanding and management of various health conditions. The integration of multi-omics techniques, including metatranscriptomics, metaproteomics, and metabolomics, has been instrumental in advancing our knowledge of the gut microbiome and its complex interactions with human health and disease. These approaches provide a more comprehensive understanding of the gut microbiome by capturing not only the taxonomic composition, but also the functional activities, metabolic processes, and interactions within the microbial community. Metatranscriptomics examines the RNA molecules expressed by the microbial community, revealing insights into their functional activities and gene expression patterns. Metaproteomics focuses on identifying and quantifying the proteins expressed by the gut microbiome, providing valuable information about their metabolic activities and functional capabilities, as well as the interactions between the microbiome and the host. Furthermore, metabolomics, the study of small-molecule metabolites, can uncover the complex interplay between the microbial community and the host's physiology, identifying metabolic pathways and biomarkers associated with various health and disease states. The integration of these multi-omics approaches has enabled researchers to unravel the complex relationships between the gut microbiome and human health, leading to the identification of novel biomarkers and the development of predictive models for personalized medicine.
